# Supplementary material for: Mechanisms governing the pioneering and redistribution capabilities of the non-classical pioneer PU.1
Source: Nat Commun. 2020 Jan 21;11:402. doi: 10.1038/s41467-019-13960-2 (PMC6972792; doi:10.1038/s41467-019-13960-2)
Supplement: Supplementary file 7 — Source data [file 41467_2019_13960_MOESM7_ESM.zip › Source_Data/FigureS2/FigureS2B_MotifScanOutput/KmeansCluster6/homerResults/motif29.similar.html]

motif29

## Information for motif29

A
G
C
T
C
T
A
G
A
G
T
C
A
G
T
C
C
G
T
A
A
G
C
T
T
C
A
G
C
G
A
T
C
T
A
G
C
G
T
A
  
Reverse Opposite:  

G
C
A
T
A
G
T
C
G
C
T
A
A
G
T
C
C
T
G
A
G
C
A
T
C
T
A
G
T
C
A
G
A
G
T
C
T
C
G
A
  

|  |  |
| --- | --- |
| p-value: | 1e-41 |
| log p-value: | -9.467e+01 |
| Information Content per bp: | 1.623 |
| Number of Target Sequences with motif | 359.0 |
| Percentage of Target Sequences with motif | 10.90% |
| Number of Background Sequences with motif | 2058.4 |
| Percentage of Background Sequences with motif | 4.79% |
| Average Position of motif in Targets | 98.3 +/- 52.8bp |
| Average Position of motif in Background | 101.1 +/- 65.7bp |
| Strand Bias (log2 ratio + to - strand density) | -0.1 |
| Multiplicity (# of sites on avg that occur together) | 1.09 |
| Motif File: | file (matrix) reverse opposite |

### Similar de novo motifs found

|  |  |  |  |  |  |  |  |
| --- | --- | --- | --- | --- | --- | --- | --- |
| Rank | Match Score | Redundant Motif | P-value | log P-value | % of Targets | % of Background | Motif file |
| 1 | 0.825 | G A C T G A T C G T C A A G T C C T G A C G A T C T A G C T A G A G T C C T A G T C A G | 1e-33 | -76.800965 | 4.25% | 1.14% | motif file (matrix) |
| 2 | 0.750 | C G A T C T A G A G T C A G T C C T G A A G C T A C T G | 1e-13 | -30.330293 | 16.79% | 12.17% | motif file (matrix) |
